# Perspiration during Exertion

**Published:** 1901-08-31

**Authors:** 


### PERSPIRATION DURING EXERTION.

Of course we all know how useful a good diaphoresis may be when from any cause the excretion of nitrogenous material by the kidneys is interfered with; but we doubt if many of us quite recognise how very large a proportion of the total nitrogen excreted passes off by the skin when free sweating takes place as a result of exercise. Certain observations made by Drs. Zuntz and Schumberg in regard to the metabolism of soldiers on the march have brought to light some very interesting facts as to the effects of steady exercise. Among other things investigated were the amount and the character of the perspiration. The soldiers under examination were clothed in specially washed garments, and after the march was over, when the amount of nitrogen absorbed by these clothes was estimated, it was found that as much as 12 per cent. of the total amount of nitrogen given off in the urine and the fæces might be excreted in the sweat during hard work. It is important to note that increase in this output in the sweat seemed to be related to heat of weather rather than to increase of load. The bearing of this on the question of clothing is sufficiently obvious. Perspiration is a part of the heat regulating mechanism, and unless the sweat can evaporate it fails in its object. Clothing should therefore be porous so as to allow of evaporation. That we have always recognised. But what is particularly shown by these researches is the very large amount of nitrogenous waste which is contained in this perspiration, waste which does not evaporate, but remains in the pores of the clothes, rendering them offensive and dangerous. Not only then must the clothes worn by those undergoing exertion be porous to allow of evaporation, but they must be capable of being frequently washed Otherwise the unfortunate man will walk about clothed in his own excreta—as we fear too many people do.

